# A Comparative Study of Scientific Publications in Health Care Sciences and Services from Mainland China, Taiwan, Japan, and India (2007–2014)

**DOI:** 10.3390/ijerph13010079

**Published:** 2015-12-24

**Authors:** Yipeng Lv, Bihan Tang, Xu Liu, Chen Xue, Yuan Liu, Peng Kang, Lulu Zhang

**Affiliations:** Institute of Military Health Management, Second Military Medical University, 800 Xiangyin Rd, Shanghai 200433, China; epengl@163.com (Y.L.); mangotangbihan@126.com (B.T.); aqualau@126.com (X.L.); xuechen8990@163.com (C.X.); yawnlau@126.com (Y.L.); kpkp315@163.com (P.K.)

**Keywords:** health care sciences and service, impact factor, Science Citation Index Expanded

## Abstract

In this study, we aimed to compare the quantity and quality of publications in health care sciences and services journals from the Chinese mainland, Taiwan, Japan, and India. Journals in this category of the Science Citation Index Expanded were included in the study. Scientific papers were retrieved from the Web of Science online database. Quality was measured according to impact factor, citation of articles, number of articles published in top 10 journals, and the 10 most popular journals by country (area). In the field of health care sciences and services, the annual incremental rates of scientific articles published from 2007 to 2014 were higher than rates of published scientific articles in all fields. Researchers from the Chinese mainland published the most original articles and reviews and had the highest accumulated impact factors, highest total article citations, and highest average citation. Publications from India had the highest average impact factor. In the field of health care sciences and services, China has made remarkable progress during the past eight years in the annual number and percentage of scientific publications. Yet, there is room for improvement in the quantity and quality of such articles.

## 1. Introduction

Public and professional interest in health care sciences and services has increased dramatically over the last decade. Research in the health care sciences has contributed to a better comprehension of health determinants and to the development of preventative and curative interventions. Knowledge and understanding of health service usage are necessary for health resource allocation, planning, and monitoring for achievement of universal coverage [1].

Numbering more than 1.3 billion, people of Chinese ethnicity make up the largest population in the world. India—with more than 1.2 billion people—ranks second; it is one of the most productive Asian countries in the realm of scientific research [2]. Japan—as a neighboring country to China—is also among the top-ranking countries in scientific research [3]. The study of scientific publications in a particular field—based on international bibliographic data—is one of the most widely used methods for measuring scientific achievement [4]. In the past decade, we have witnessed the remarkable development of China in scientific research publications; the country has ranked second in total annual scientific publications since 2006 [5]. The Chinese government launched its greatest health service system reform in 2009, which brought many changes to this ancient land. However, little is known yet about Chinese authors’ scientific contributions in the field of health care sciences and services. Furthermore, a comprehensive comparison between countries (areas) in Asia has not been undertaken.

In this study, we sought to evaluate the quantity and quality of scientific publications in the field of health care sciences and services among Chinese authors from 2007 to 2014; further, we compared these with publications from Japan and India. The Web of Science database divides Chinese authors into two categories: those from the Chinese mainland (ML) and those from Taiwan (TW). As a result, we discuss four areas in this article: ML, TW, Japan (JPN), and India (IND).

## 2. Materials and Methods

In this retrospective study, we examined 89 journals related to health care sciences and services selected from this category of the Science Citation Index Expanded (SCIE) provided by the Institute for Scientific Information (ISI) [6]. The category covers resources related to health services, hospital administration, health care management, health care financing, health policy and planning, health economics, health education, the history of medicine, and palliative care [7]. In such research, the “scientific publications” refers to all kinds of papers published in the journal based on the web of science including article, news, case report, bibliography, letter, correction review, biography, reference material, report, abstract, clinical trial, book, government publication editorial, meeting, legislation and others. A computerized literature search was conducted using the Web of Science database on 10 September 2015. At first, we aimed to investigate scientific publications regarding health care sciences and services during the past 10 years; however, there was no information in the Web of Science database for “country categories” for articles written before 2007. As a result, we examined scientific publications in the field of health care sciences and services that appeared between 1 January 2007, and 31 December 2014. The full names of the 89 journals were used to perform this study search. Scientific output from the four countries (areas) was identified using the categories in the Web of Science database. Original articles and reviews were compiled using the publication categories in the Web of Science database.

Five methods were used to compare the quality of research articles. First, the accumulated and average impact factors (IFs) were generated according to the Journal Citation Reports (JCR) 2014, a database established by ISI containing information about the number of times each year that a journal is cited and the name of the citing journal [8]. The JCR has been published by the ISI since 1975; it is considered the most comprehensive citation index of scientific literature [9], covering more than 8000 journals in 2014 [8]. Second, we quantified the citations of articles written by researchers from the four countries (areas). Third, we calculated the number of original articles and reviews that were associated with a higher grade of evidence. (In the Web of Science database, the term “original article” refers to “reports of research on original works,” and “review” refers to “a renewed study of material previously studied.”) [10]. Fourth, the number of articles published by researchers from each country (area) in the top 10 high-impact health care sciences and services journals was also compared. Finally, we determined the top 10 most popular health care sciences and services journals for the four countries (areas) according to the number of articles published by each journal.

### Statistical Analyses

All data were analyzed using the Statistical Package for Social Sciences (SPSS version 21.0, SPSS Inc., Chicago, IL, USA, 2012). The annual incremental rate was used to reflect the changing trends in scientific publications from 2007 to 2014. A linear correlation analysis with coefficient “r” was performed to determine any significant change in total numbers over the given period of time. The Kruskal-Wallis test was used to detect differences among the four countries (areas), and Nemenyi tests were conducted to detect the differences between two countries (areas) when necessary. All statistical tests were two-sided, and *p* < 0.05 was considered to be of statistical significance. The Ethics Committee of the Second Military Medical University approved the procedures and methods used in this study.

## 3. Results

### 3.1. Total Number of Scientific Articles

In total, 20,252,178 scientific articles were published in SCIE-cited journals from 2007 to 2014 worldwide: 6.7% were from ML (rank 2), 1.1% from TW (rank 17), 3.7% from JPN (rank 5), and 2.0% from IND (rank 11). The annual number of published scientific articles increased significantly from 2007 to 2014 in ML, TW, JPN, and IND (Table 1).

### 3.2. Number of Articles in the Field of Health Care Sciences and Services

**Figure 1:**
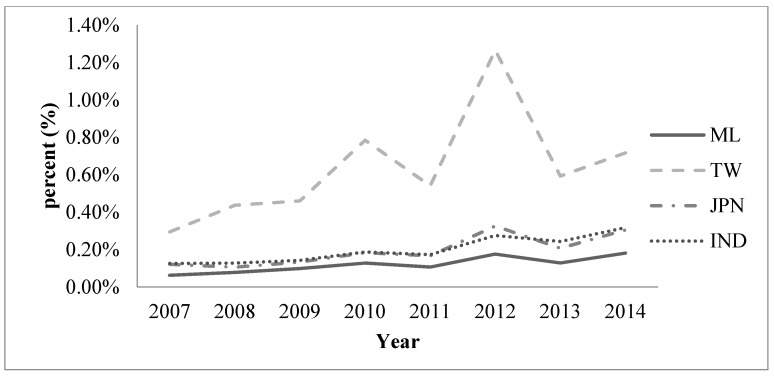
Percentage of publications in the area of health care sciences and services within all scientific publications by researchers from Mainland China (ML), Japan (JPN), Taiwan (TW), and India (IND) (2007–2014).

In total, 149,957 scientific articles were published in the 89 selected journals from 2007 to 2014. Among these, 1772 publications in SCIE-cited journals were from ML; 1397 were from TW; 1450 were from JPN; and 848 were from IND. Health care sciences and services articles comprised 0.13% of the total number of scientific publications in ML, 0.66% in TW, 0.19% in JPN, and 0.21% in IND. The annual number of published scientific articles in the field of health care sciences and services increased significantly from 2007 to 2014 in ML, TW, JPN, and IND (Table 1). From 2007 to 2014, ML contributed 1.18% of the total global output in the field of health care sciences and services papers and ranked 12th; TW contributed 0.93% and ranked 17th; JPN contributed 0.97% and ranked 16th; and IND contributed 0.57% and ranked 21st (Table 1 and Figure 1).

**Table 1:** Annual numbers of scientific articles and articles in the field of “health care sciences and services” published by researchers from Mainland China (ML), Japan (JPN), Taiwan (TW), and India (IND) (2007–2014).

Year	Scientific Articles				Articles in the Field of “Health Care Sciences and Services”			
	ML	TW	JPN	IND	ML	TW	JAN	IND
2007	99,640	21,025	91,109	36,474	62	62	111	46
2008	115,166	23,569	91,234	42,784	90	103	97	55
2009	132,058	25,412	92,679	44,141	130	117	124	63
2010	146,818	26,482	91,583	47,503	188	208	168	89
2011	169,866	28,268	92,333	51,791	181	153	154	90
2012	195,202	29,149	93,993	54,748	343	369	307	151
2013	233,031	29,477	96,362	60,687	299	175	200	148
2014	264,429	29,271	94,609	64,745	479	210	289	206
Total	135,6210	212,653	743,902	402,873	1772	1397	1450	848
Rank in the world	2nd	17th	5th	12th	12th	17th	16th	21st
r Value	0.986	0.937	0.845	0.993	0.945	0.651	0.844	0.954
Incremental rate	20.67%	4.90%	0.48%	9.69%	84.07%	29.84%	20.05%	43.48%

### 3.3. Types of Articles in the Field of Health Care Sciences and Services

As shown in Table 2, researchers from ML published more “original articles” than researchers from TW, JPN, and IND (ML > TW> JPN > IND); such articles accounted for 55.64%, 60.84%, 57.72%, and 43.99% of the total number of articles in the field of health care sciences and services in each country (area), respectively. Furthermore, researchers from ML published more “reviews” than researchers from JPN, IND, and TW (ML > JPN> IND> TW), accounting for 3.10%, 2.69%, 1.77%, and 0.93% of the total number of articles in the field of health care sciences and services of each country (area).

**Table 2:** Numbers of original articles and reviews published by researchers from Chinese Mainland (ML), Japan (JPN), Taiwan (TW), and India (IND) (2007–2014).

Type of Paper	ML (%)	TW (%)	JPN (%)	IND (%)	Total (%)
Article	986 (55.64%)	850 (60.84%)	837 (57.72%)	373 (43.99%)	3046 (55.72%)
Review	55 (3.10%)	13 (0.93%)	39 (2.69%)	15 (1.77%)	122 (2.23%)
Total number of papers in the field of “health care sciences and services”	1772	1397	1450	848	5467

### 3.4. Impact Factors (IFs)

**Figure 2:**
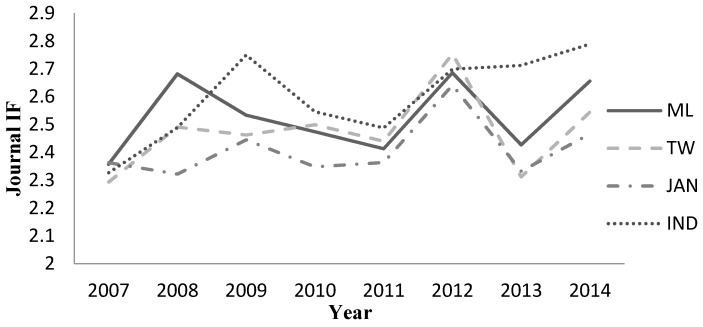
The trend in average Impact Factor (IF) of articles in the field of “health care sciences and services” published by researchers from Mainland China (ML), Japan (JPN), Taiwan (TW), and India (IND) (2007–2014).

The IFs indicate the average number of article citations in publications. According to the JCR, 86 of the health care sciences and services journals had IFs in 2014, and the following three journals did not: *Eastern Mediterranean Health Journal*, *Journal of the American Association of Nurse Practitioners*, and *Quality Management in Health Care*. The accumulated IFs of articles from ML, TW, JPN, and IND differ from each other significantly (4535.3, 3535.4, 3539.4, and 2251.2, respectively; F = 60.848, *p* < 0.0001). The average IF of articles from IND (2.97) was higher than that of articles from ML (2.82), TW (2.77), and JPN (2.71) (F = 12.77, *p* < 0.0001). Figure 2 shows the trend in the average IF of articles in the field of health care sciences and services published by researchers from PRC, JPN, TW, and IND (2007–2014).

### 3.5. Citations of Articles Published in Health Care Sciences and Services Journals

Articles from ML were the most cited (7729 citations), followed by those from TW (5469 citations), JPN (5350 citations), and IND (2536 citations). The average number of citations of each article from ML, TW, JPN, and IND was 4.4, 3.9, 3.7, and 3.0, respectively. The highest number of citations of articles was from ML (145). TW, JPN, and IND reached 119, 109, and 65, respectively.

### 3.6. Articles in the 10 Top-Ranking Health Care Sciences and Services Journals

A total of 569 articles from the four countries (areas) were published in the 10 top-ranking health care sciences and services journals. Among them, 11.2% (64/569) were published in the top three journals (*Health Technology Assessment*, *Health Affairs* and *Statistical Methods in Medical Research*). Researchers from ML published 153 (26.9%) articles in 10 high-impact health care sciences and services journals, whereas those from TW published 111 (19.5%); those from JPN published 216 (38.0%); and those from IND published 89 (15.6%).

### 3.7. Popular Health Care Sciences and Services Journals

The journals that published the most articles by researchers from the four countries (areas) studied are listed in Table 3. *Value in Health* was the most popular journal among all four countries (areas) (Table 4). The top three journals with the most articles from TW, JPN, and IND are the same—*Value in Health*, *Therapeutics and Clinical Risk Management*, and *Telemedicine and E-Health*. The sums of IFs of the 10 journals publishing the most papers from ML, TW, JPN, and IND were 22.67, 21.05, 19.43, and 21.45, respectively.

## 4. Discussion

India (IND), JPN, ML, and TW are all major countries (areas) in the world in terms of population, economy, and scientific research. As one of the world’s most developed countries, JPN has been leading global scientific research for decades. Moreover, IND, ML, and TW have made great advances in the past few decades, with rapid development in education, urbanization, economy, and scientific research [11].

The number of articles published in scientific journals is a reflection of research activity in a given country or area [12,13]. The results of this study showed that both the total number and percentage of scientific publications from ML were the highest in Asia. Therefore, there is no doubt that ML leads all other Asian countries (areas) in scientific publication productivity. The active role of ML in scientific research may be attributed to the following factors: major improvements in policy reform, a meteoric economic rise, the modernization of diagnostic and therapeutic methods, an increase in the number of researchers and research funds, and more frequent international collaboration [14,15]. Government research funds in China have been growing at an annual rate of more than 20%—exceeding even the expectations of China’s most enthusiastic scientists [16]. Indeed, the Chinese government has placed a greater emphasis on scientific development than ever before. However, some observers and critics have suggested that a Chinese researcher's funding is based on the number of papers listed in the Thomson Reuters Science Citation Index and the IFs of the journals in which they are published, rather than on an evaluation of a work’s scientific importance [17]. This distribution rule may lead to unfair resource allocation, benefiting the advantage discipline and hindering the inferior discipline.

**Table 3:** Papers published in the top 10 popular health care sciences and services journals by researchers from Mainland China (ML), Japan (JPN), Taiwan (TW), and India (IND) (2007–2014).

Rank	Journal	ISSN	ML	TW	JPN	IND	Total	IF
1	*VALUE IN HEALTH*	1098–3015	572	407	257	339	1575	3.279
2	*JOURNAL OF MEDICAL SYSTEMS*	0148–5598	113	162	53	89	417	2.213
3	*QUALITY OF LIFE RESEARCH*	0962–9343	128	77	114	18	337	2.486
4	*BMC HEALTH SERVICES RESEARCH*	1472–6963	116	79	54	34	283	1.712
5	*JOURNAL OF GENERAL INTERNAL MEDICINE*	0884–8734	37	61	170	14	282	3.449
6	*HEALTH POLICY*	0197–5897	72	44	72	54	242	1.907
7	*SUPPORTIVE CARE IN CANCER*	0941–4355	68	50	101	13	232	2.364
8	*JOURNAL OF PAIN AND SYMPTOM MANAGEMENT*	0885–3924	25	32	67	5	129	2.795
9	*MEDICAL TEACHER*	0142–159X	56	22	23	22	123	1.679
10	*JOURNAL OF PALLIATIVE MEDICINE*	1096–6218	14	14	63	19	110	1.912
	TOTAL NUMBER		1201	948	974	607	3730	

**Table 4:** Top 10 journals publishing the most articles written by researchers from Mainland China (ML), Japan (JPN), Taiwan (TW), and India (IND).

ML	IF	N	TW	IF	N	JPN	IF	N	IND	IF	N
*Value Health*	3.279	572	*Value Health*	3.279	407	*Value Health*	3.279	257	*Value Health*	3.279	339
*Qual. Life Res.*	2.486	128	*Ther. Clin. Risk. Manag.*	1.469	1	*Ther. Clin. Risk. Manag.*	1.469	23	*Ther. Clin. Risk Manag.*	1.469	4
*BMC Health Serv. Res.*	1.712	116	*J. E-Health*	1.668	24	*J. E-Health*	1.668	8	*J. E-Health*	1.668	9
*J. Marine Systs.*	2.213	113	*Technol. Health Care*	0.697	9	*Technol. Health Care*	0.697	13	*Technol. Health Care*	0.697	17
*Support Care Cancer*	2.364	68	*Teach. Learn Med.*	0.659	1	*Teach. Learn Med.*	0.659	3	*Teach. Learn Med.*	0.659	2
*Med. Teach.*	1.679	56	*Support Care Cancer*	2.364	50	*Support Care Cancer*	2.364	101	*Support Care Cancer*	2.364	13
*Health Qual. Life Out*	2.12	55	*Stat. Methods Med. Res.*	4.472	1	*Qual. Life Res.*	2.486	114	*Stat. Methods Med. Res.*	4.472	4
*Ther. Clin. Risk Manag.*	1.469	44	*Qual. Life Res.*	2.486	77	*Popul. Health Manag.*	1.509	2	*Qual. Life Res.*	2.486	18
*J. Gen. Intern. Med.*	3.449	37	*Popul. Health Manag.*	1.509	4	*Pharmacoeconomics*	2.45	3	*Popul. Health Manag.*	1.509	2
*Health Policy*	1.907	35	*Pharmacoeconomics*	2.45	5	*J. Palliat. Med.*	2.855	12	*J. Palliat. Med.*	2.855	2
Total	22.678	1224		21.053	579		19.436	536		21.458	410

Fortunately, this situation has changed gradually since 2012, greatly promoting the development of inferior disciplines such as the health care sciences and services. The incremental rate of articles published by researches from ML in the field of health care sciences and services is much higher than the incremental rate of articles in all scientific fields of ML. This phenomenon reflects researchers’ increasing interest in the field of health care services and sciences. The Chinese government started their medical and health service system reform in 2009 to achieve more affordable national health care. Thus, health care sciences and services involve more than surgery to cure a person with an acute disease; now, they include preventive care, which may be the key to lowering costs. Although health care science is a relatively new field in global medicine, China, especially, has realized important advances since this field came into focus 10 years ago. The annual number of health care sciences and services articles from ML has increased significantly, surpassing that of JPN in 2009.

However, it cannot be denied that compared with the USA, ML has much room for development in this area. A database search revealed that the USA publishes the most articles in the field of health care sciences and services, accounting for 35.53% of total articles. Furthermore, articles in this field published by USA researchers accounted for 1.5% of all articles in all fields published from 2007 to 2014. It should be noted that the share of health care sciences and service publications within the total number of scientific publications was the smallest (0.13%) in ML.

There are several explanations for the low proportion of scientific publications in the health care sciences and services field in ML. First, there is a large disparity between the urban and rural areas. Although ML has made great achievements in its economy in the past decade, most of the rural population still lives in poverty. More than 50% of the rural population cannot afford any kind of medical care [18,19]. Thus, health care sciences and services development is at a relatively low level in rural areas—far from the modernization level associated with the publication of scientific articles in international journals. Second, the use of English as the language of publication for most scientific publications is a problem for researchers in ML. Compared to researchers in TW, a substantial number of researchers in ML are more accustomed to publishing their studies in Chinese journals.

The IF is known as a significant scientometric parameter used for assessment of the quality of individual papers, scientists, and departments [20]. Our study showed that the differences between the four countries (area) examined were significant in terms of average IFs (*p* < 0.05). As shown by the results of the study, ML has a high average IF among the four countries (areas), second only to IND. Further, there is no significance between ML and TW. Additionally, IFs of the four countries (areas) fluctuated continuously from 2007 to 2014 (Figure 2). In 2007, the difference between the four countries (areas) was not significant, and the gap widened in 2014. Although IF is not an appropriate measure of the scientific quality of individual articles [21], it is still one of the most useful tools with which to gauge the relative importance of scientific researches [22]. Thus, compared with ML, articles in the field of health care sciences and services published by researchers from IND are of higher quality overall despite the smaller quantity.

**Figure 3:**
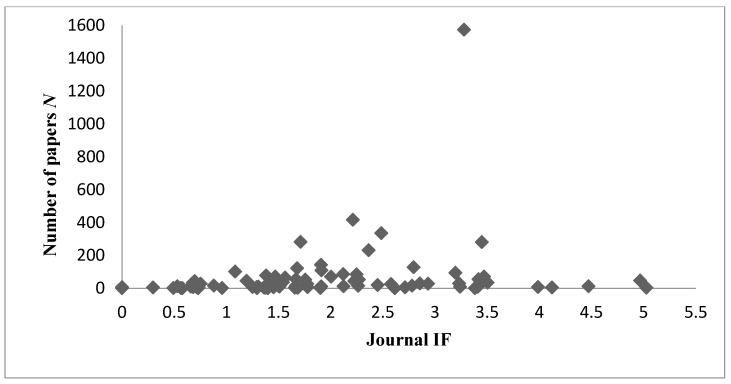
Number N of papers published in 89 journals in the field of health care sciences and services as a function of their impact factors (IFs) during 2007–2014.

Another important indicator of article quality is the analysis of citation indices. The number of citations that an article receives reflects its scientific impact to some extent [23]. Our study demonstrated that articles from ML had the most citations and highest average for citations. Furthermore, ML has published the most influential articles in the field. Regarding the top 10 health care sciences and services journals, researchers from ML published more papers in these than researchers from TW and IND; only JPN published more articles in the target journals. In summary, our comparison of publication quality using IFs, the citation index, number of original articles, number of reviews, and number of articles published in the top 10 journals demonstrated that ML was leading in the health care sciences and services field in Asia as of 2014. The results also indicate that the IFs of the 10 most popular health care sciences and services journals of these four countries (areas) were in the middle range of the ranking of IFs in all related journals (Figure 3).

There are some inherent limitations to this study. First, the journals used were selected from the health care sciences and services category of the 2015 SCIE. Some of the journals included in this database change each year, though most of the journals remained unchanged. In addition, some relevant journals were not included in the health care sciences and services category of the SCIE. Second, accumulated IFs and the average IFs were evaluated by utilizing the IFs of JCR 2015. In the past decade, IFs of the journals changed from year to year. The accumulated IFs and average IFs reported in this study are therefore only estimations, but they are likely to reflect this trend because alterations in IFs have been relatively small for most journals in the past eight years. Despite these limitations, we believe that the results of this study are likely to reflect the real situation of health care sciences and services research in ML, TW, JPN, and IND.

## 5. Conclusions

In summary, our study demonstrated that ML ranked first in Asia in 2014 for the total number of scientific articles published. In the field of health care sciences and services, ML has made remarkable progress in the annual number and percentage of scientific publications during the past eight years (2007–2014). Nevertheless, there is still room for improvement in the quantity and quality of health care sciences and services articles. Therefore, effective measures should be taken to promote scientific studies in the health care sciences and services field by researchers in ML.
